# Reparative effect of mesenchymal stromal cells on endothelial cells after hypoxic and inflammatory injury

**DOI:** 10.1186/s13287-020-01869-3

**Published:** 2020-08-12

**Authors:** Jesus M. Sierra-Parraga, Ana Merino, Marco Eijken, Henri Leuvenink, Rutger Ploeg, Bjarne K. Møller, Bente Jespersen, Carla C. Baan, Martin J. Hoogduijn

**Affiliations:** 1 Internal Medicine Department, Sector Nephrology & Transplantation, University Medical Center Rotterdam, Erasmus MC, Postbus 2040, 3000 CA Rotterdam, the Netherlands; 2 Department of Renal Medicine, Aarhus University Hospital, Aarhus, Denmark; 3 Department of Clinical Immunology, Aarhus University Hospital, Aarhus, Denmark; 4 Department of Surgery – Organ Donation and Transplantation, University Medical Center Groningen, University of Groningen, Groningen, the Netherlands; 5 Nuffield Department of Surgical Sciences and Oxford Biomedical Research Centre, University of Oxford, Oxford, UK; 6 Department of Clinical Medicine, Aarhus University, Aarhus, Denmark

**Keywords:** Mesenchymal stromal cells (MSC), Ischemia-reperfusion-injury (IRI), Tissue repair, Angiogenesis, Endothelium

## Abstract

**Background:**

The renal endothelium is a prime target for ischemia-reperfusion injury (IRI) during donation and transplantation procedures. Mesenchymal stromal cells (MSC) have been shown to ameliorate kidney function after IRI. However, whether this involves repair of the endothelium is not clear. Therefore, our objective is to study potential regenerative effects of MSC on injured endothelial cells and to identify the molecular mechanisms involved.

**Methods:**

Human umbilical vein endothelial cells (HUVEC) were submitted to hypoxia and reoxygenation and TNF-α treatment. To determine whether physical interaction or soluble factors released by MSC were responsible for the potential regenerative effects of MSC on endothelial cells, dose-response experiments were performed in co-culture and transwell conditions and with secretome-deficient MSC.

**Results:**

MSC showed increased migration and adhesion to injured HUVEC, mediated by CD29 and CD44 on the MSC membrane. MSC decreased membrane injury marker expression, oxidative stress levels, and monolayer permeability of injured HUVEC, which was observed only when allowing both physical and paracrine interaction between MSC and HUVEC. Furthermore, viable MSC in direct contact with injured HUVEC improved wound healing capacity by 45% and completely restored their angiogenic capacity. In addition, MSC exhibited an increased ability to migrate through an injured HUVEC monolayer compared to non-injured HUVEC in vitro.

**Conclusions:**

These results show that MSC have regenerative effects on injured HUVEC via a mechanism which requires both physical and paracrine interaction. The identification of specific effector molecules involved in MSC-HUVEC interaction will allow targeted modification of MSC to apply and enhance the therapeutic effects of MSC in IRI.

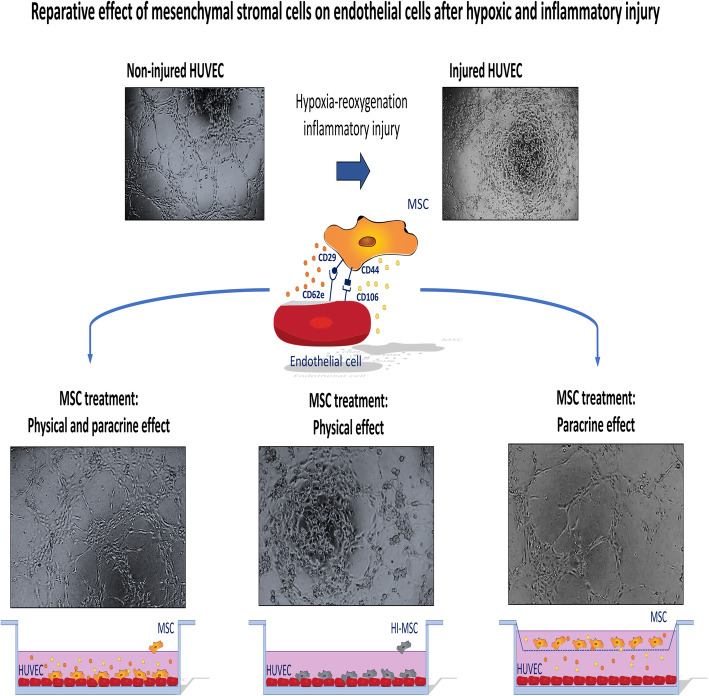

## Background

Ischemia-reperfusion injury (IRI) of a transplanted organ cannot be avoided and initiates an inflammatory cascade leading to organ dysfunction [1].

IRI especially impacts organs from donation after circulatory death and from expanded criteria donors with age higher than 60 years comorbidities such as hypertension [2]. The increasing use of organs from these donors enables enlarging the donor organ pool, but these organs tend to be more sensitive to injury which is associated with worse transplantation outcome [3, 4]. IRI particularly leads to endothelial injury, resulting in their activation and the loss of endothelial wall integrity and function [5–8].

Mesenchymal stromal cells (MSC) may represent a unique cellular tool to repair damaged endothelium. MSC are adult multipotent cells present in most tissues in the adult human body [9, 10]. These cells are known for their regenerative and anti-inflammatory properties, which have been explored in a number of small animal [11–13] and large animal [14–16] injury models. In these experimental models, MSC have been shown to have regenerative properties that may promote endothelial repair. For this reason, MSC are being studied as a therapeutic agent for kidney disease and transplantation in man [17]. Several phase I trials have shown that MSC therapy is safe and suggest that their immunoregulatory and regenerative properties might lead to an improved kidney transplantation outcome [18–21]. In most of these studies, the MSC were given via intravenous (IV) infusion which led to entrapment of MSC in the pulmonary capillaries that prevented MSC delivery to the injured kidney [22]. Thus, direct MSC infusion to the injured tissue may be a better method to deliver MSC to the kidney. We have previously shown that for infusion via the renal artery, MSC are delivered to the microvasculature of the kidney [23]. The interaction with the kidney microvasculature is the starting point for the regenerative effects of MSC, and therefore, it is of key importance to understand the mechanisms of the interaction between MSC and endothelial cells. A better understanding of these interactions will enable us to optimize the potential regenerative effect of MSC therapy on endothelial injury. The first data about MSC delivery to the kidney during ex situ normothermic machine perfusion show a distribution of MSC to the renal microvasculature, with MSC localizing to the renal cortex [24]. Although data suggest that MSC may possess renal regenerating effects after administration to the kidney [25], it is unknown whether this involves endothelial repair, and if so which mechanisms are involved.

Here, we investigated the potential reparative effects of MSC on endothelial cells, which were subjected to hypoxic and inflammatory insults. We tested whether physical or paracrine interaction of MSC and endothelial cells was responsible for the regenerative effects of MSC and which molecules were involved.

## Methods

### Isolation and culture of MSC

MSC were isolated from subcutaneous adipose tissue from healthy human kidney donors (*n* = 5) that became available during kidney donation procedures after obtaining written informed consent as approved by the Medical Ethical Committee of the Erasmus University Medical Centre Rotterdam (MEC-2006-190).

MSC were cultured at 37 °C, 5% CO_2_, and 20% O_2_ in minimum essential medium-α (Sigma Aldrich, St. Louis, MO, USA) supplemented with penicillin (100 IU/ml), streptomycin (100 mg/ml) (1% P/S; Lonza), 2 mM l-glutamine (Lonza), and 15% fetal bovine serum (Lonza) in T175 flasks (Greiner Bio-one, Kremsmunster, Austria). Culture medium was replaced twice a week. MSC were used at passage 3–6.

### Culture of HUVEC

Human umbilical vein endothelial cells (HUVEC) were purchased from Lonza (Basel, Switzerland) and cultured at 37 °C, 5% CO_2_, and 20% O_2_ in endothelial growth medium 2 (EGM-2, Lonza) supplemented with 5% heat-inactivated fetal bovine serum in T75 culture flasks (Greiner Bio-one). Culture medium was replaced three times a week. HUVEC were used at passage 3–6.

### Immunophenotyping of MSC and HUVEC

HUVEC and MSC were phenotyped based on the expression of specific molecules on their cell surface. Damage and activation markers were also measured on the surface of HUVEC and MSC by flow cytometry (FACS Canto II, BD Biosciences, NJ, USA). Monoclonal antibodies conjugated with different fluorophores were used to measure the presence of these molecules.

Markers measured on MSC surface membranes were CD29 (Cat# 11-0299-42, eBioscience, Santa Clara, CA, USA), CD31 (Cat#555445, Becton Dickinson), CD38 (Cat# 562444, Becton Dickinson), CD44 (Cat# 553134, Becton Dickinson), CD45 (Cat#345809, Becton Dickinson), CD54 (Cat#559771, Becton Dickinson), CD62e (Cat#551145, Becton Dickinson), CD73 (Cat# 550257, Becton Dickinson), CD144 (Cat# 348510, Biolegend, San Diego, CA, USA), CD146 (Cat#747737, Becton Dickinson), TGF-β rII (Cat#FAB241P, R&D Systems, Minneapolis, MN, USA), PD-L1 (Cat# 557924, Becton Dickinson), and HLA-II (Cat#347402, Becton Dickinson).

Markers measured on HUVEC membrane were CD31 (Cat#555445, Becton Dickinson), CD54 (Cat#559771, Becton Dickinson), CD62e (Cat#551145, Becton Dickinson), CD105 (Cat# FAB10971F, R&D Systems), CD106 (Cat#744309, Becton Dickinson), CD144, (Cat# 348510, Biolegend, San Diego, CA, USA), CD146 (Cat#747737, Becton Dickinson), Tie-2 (Cat# FAB3131N, R&D Systems), HLA-II (Cat#347402, Becton Dickinson), and VEGF-r2 (Cat#560494, Becton Dickinson). Data were analyzed using Kaluza Analysis 2.1 (Beckman Coulter).

### In vitro hypoxia-reoxygenation injury model

HUVEC were trypsinized from the culture flask cells, washed with phosphate-buffered saline (PBS), and detached using 2 ml 0.05% Trypsin-EDTA (ThermoFisher) for 2 min. HUVEC were seeded at a concentration of 3 × 10^4^ cells/cm^2^ and cultured until complete confluence in EGM-2 medium (Lonza) at 37 °C, 5% CO_2_, and 20% O_2_ in culture wells. Oxygen was enzymatically removed from culture medium using bovine catalase (0.43 mg/ml, Sigma) and glucose oxidase (0.125 mg/ml, Sigma) as previously described [26] to quickly remove all oxygen from the medium. Oxygen percentage was measured using a universal perfusion solution monitor (version 1.10, Hugo Sachs Elektronik -harvard Apparatus GmbH, March-Hugstetten, Germany). Culture medium was removed, and cells were washed with PBS prior to the addition of hypoxic medium. Hypoxia was maintained by culturing HUVEC in a hypoxia incubator during 24 h at 37 °C, 5% CO_2_, and 0–1% O_2._ Additional file 1A shows that levels of oxygen were around 0% from the addition of the catalase and glucose oxidase and until O_2_ measurement after 24 h. After this time point, cultures were washed with PBS and normoxic culture medium supplemented with human recombinant 20 ng/ml tumor necrosis factor-α (TNF-α; Peprotech, Rocky Hill, NJ, USA) was added for 24 h to mimic the inflammatory response after ischemia-reperfusion (injured HUVEC). All experiments described below were performed five times.

### HUVEC-MSC incubation

After culture of HUVEC in normoxic conditions with TNF-α, culture medium was removed and wells were washed with PBS. Fresh HUVEC medium without TNF-α was then added to HUVEC. MSC were resuspended in MSC medium and added to HUVEC. The resulting medium was a 1:1 MSC medium-HUVEC medium ratio used for the co-culture of MSC and HUVEC. The final serum ratio was 10%. To assess MSC effect on HUVEC injury generated by hypoxic and inflammatory conditions, MSC were added in an MSC-HUVEC ratio of 1:2 or 1:10. In order to study the effect of MSC on HUVEC, MSC were added to HUVEC in three different manners (Additional file 1D-F). Firstly, MSC were directly incubated with HUVEC for 24 h after injuring HUVEC, allowing cell-to-cell physical and paracrine communication. Secondly, to determine the effect of physical MSC-HUVEC interaction, MSC were inactivated by warming them to 50 °C during 30 min as previously described [22]. After this procedure, the metabolism of MSC is completely stopped and they lose their ability to secrete soluble factors, but the cell surface membrane and its associated proteins remain intact. Lastly, to assess the effect of cytokines and growth factor released by MSC on HUVEC, MSC were incubated with HUVEC in a transwell system. The transwell had a porous membrane of pore size 0.4 μm (Greiner Bio-One) that allows communication through soluble factors but prevents physical contact or cell migration. In all three conditions, the incubation of MSC with HUVEC started 24 h after reoxygenation and culture with TNF-α to test their reparative role.

### Assessment of HUVEC viability

HUVEC viability was assessed by Annexin-V (PE) and ViaProbe (PercP) staining using the Annexin-V apoptosis detection kit I (Becton Dickinson, Franklin Lakes, NJ, USA) and measured by flow cytometry (Additional file 1B and C). Data were analyzed using Kaluza Analysis 2.1 software (Beckman Coulter, Brea, CA, USA).

### Measurement of LDH release

HUVEC vitality was measured using a colorimetric assay to measure lactate dehydrogenase (LDH) release to the medium as a marker for cell injury. HUVEC were cultured in 96-well plates, and an LDH activity assay kit (Sigma) was used according to the manufacturer’s protocol. The results were obtained by measuring the absorbance of the reagent that is formed at 450 nm with a spectrophotometer (Victor2, PerkinElmer, Waltham, MA, USA).

### Migration of MSC towards HUVEC monolayer

Migration of MSC towards injured HUVEC was assessed by culturing a monolayer of HUVEC in the lower well of a transwell system and injuring them as described above. MSC were added on top of a porous membrane with 3-μm pore size and cultured for 6 h. After this, both upper and lower wells were trypsinized and cells counted by flow cytometry after staining them with CD31 antibody to discriminate endothelial cells from MSC. Stromal cell-derived factor 1α (SDF-1α, 10 ng/ml) was used as a positive control for MSC migration.

### MSC adhesion to HUVEC

MSC adhesion to HUVEC was assessed under static and flow conditions. MSC were fluorescently labeled with PKH26 (Sigma) following the manufacturer’s protocol in order to enable later identification. HUVEC were injured as described above, and MSC were added on top at an MSC-HUVEC ratio of 1:2 or 1:10 and incubated for 10, 30, or 60 min. After these time points, supernatant was collected and wells were washed to eliminate all non-adherent MSC. Attached cells were trypsinized and analyzed by flow cytometry. Fluorescent signal detected by flow cytometry allowed the determination of the percentage of attached MSC by comparing this number to the initial number of added MSC.

To analyze MSC adhesion under flow conditions, HUVEC were seeded in Ibidi® μ-Slide I Luer slides (Gräfelfing, Germany) grown to complete confluence and injured as described above. Subsequently, the slide was connected to a rolling pump (REGLO Analog, Ismatec, Wertheim, Switzerland) and culture media were perfused at 37 °C at a rate of 0.77 ml/min. PKH-labeled MSC were added to the perfusion system in different fashions: one time infusion of 200,000 MSC during flow, two times infusion of 100,000 MSC each during flow, one time infusion of 200,000 MSC followed by a 10-min stop in the flow to facilitate adhesion as in the adhesion experiment under static conditions, or one time infusion of 200,000 MSC which was recirculated for 10 min. After each infusion, 10 additional minutes of flow were maintained. To analyze MSC adhesion to HUVEC during flow conditions, the slides were inspected by fluorescence microscopy to identify the adhesion of PKH-labeled MSC. To calculate the percentage of adherent MSC, the content of the slide was trypsinized and MSC were counted by flow cytometry using their fluorescence to identify them and comparing this number to the initial number of added MSC.

### MSC-EC molecular interaction mechanism

In order to assess the role of specific adhesion molecules on MSC and HUVEC interaction, two molecules on the MSC cell surface were blocked. CD29 and CD44 were blocked by incubating MSC with CD29 polyclonal antibody (Cat# AF1778, R&D Systems) and CD44 polyclonal antibody (Cat# AF3660, R&D Systems) at a concentration of 2.5 μg/10^6^ MSC for 20 min. The effective blockage of these molecules was assessed by staining MSC with the previously described CD29 and CD44 antibodies and measuring fluorescence by flow cytometry. MSC with either blocked CD29, CD44, or both were added to a monolayer of injured HUVEC for 10, 30, or 60 min. After these time points, wells were washed to eliminate all non-adherent MSC. Attached cells were trypsinized and analyzed by flow cytometry. Fluorescent signal detected by flow cytometry allowed the determination of the percentage of MSC attached.

### Measurement of oxidative stress

Oxidative stress of HUVEC was measured using CellRox reagent (ThermoFisher, Manhattan, NY, USA) according to the manufacturer’s manual. Briefly, the cell-permeant CellROX reagent enters the cell, and there, it is oxidized by ROS, exhibiting red fluorescence. The production of ROS was quantified by measuring the fluorescence of oxidized CellRox inside the cell by flow cytometry. Data were analyzed using Kaluza Analysis 2.1 (Beckman Coulter).

### Measurement of HUVEC monolayer permeability

HUVEC were grown to complete confluence on a transwell insert and cultured for 48 h to allow the formation of tight intercellular junctions. The membrane of the insert had 0.4-μm size pores, which prevented cell migration but allowed soluble factor exchange with the lower well. FITC-conjugated dextran (100 mg/ml) diluted in PBS was added to the insert and incubated for 2 h, while 200 μl of PBS was added to the lower well. HUVEC monolayer permeability was assessed by measuring the amount of FITC-conjugated dextran in the lower well after 2 h by measuring fluorescence at 515 nm with a spectrophotometer (Victor2, PerkinElmer).

### Scratch assay

A scratch assay was performed on a confluent HUVEC monolayer. HUVEC were grown on 24-well plates until complete confluence, and with the tip of a 200-μl pipette, a scratch was made from top to bottom of the well, removing the cells in this area. After 2, 6, and 24 h, pictures were taken with an Axiovert 40 C microscope (Zeiss, Oberkochen, Germany) coupled to a Zeiss CanonSLR camera (Zeiss) to observe the closing of the scratched area. The size of the scratch area was measured using the plugin MRI Wound Healing Tool for Image J (National Institutes of Health, Bethesda, MD, USA).

### Angiogenesis assay

A tube formation assay was performed to evaluate the effect of hypoxic and inflammatory injury on the angiogenic potential of HUVEC. Geltrex™ LDEV-Free Reduced Growth Factor Basement Membrane Matrix was kept at 4 °C overnight prior to the experiment to allow complete thawing. At the start of the experiment, 50 μl Geltrex was added to each well of an ice-cold 96-well plate using cold pipette tips to avoid premature Geltrex solidification. The plates were incubated for 30 min at 37 °C to allow Geltrex solidification. Cells were added to the wells in a concentration of 2 × 10^4^ per well. During 6 h, pictures were taken hourly with an Axiovert 40 C microscope (Zeiss) coupled to a Zeiss CanonSLR camera (Zeiss) to evaluate the formation of tube-like structures. To evaluate the angiogenic capacity, the total length of the tubes formed during the assay was measured. The images were analyzed by Wimasis Image Analysis (Cordoba, Spain).

### Migration of MSC through a HUVEC monolayer

This setup was modified to assess MSC transmigration through a HUVEC monolayer. HUVEC were grown on top of the porous membrane of the upper well of the transwell system. After injury, HUVEC monolayer confluence was checked by microscopy and MSC were added directly on top of the HUVEC monolayer and incubated for 6 h. During this incubation, SDF-1α (10 ng/ml) was added to the bottom well to elicit a chemotactic response for MSC. Both wells were trypsinized and cells counted by flow cytometry.

## Results

### MSC migrate and adhere to endothelial cells through CD29 and CD44

**Fig. 1:**
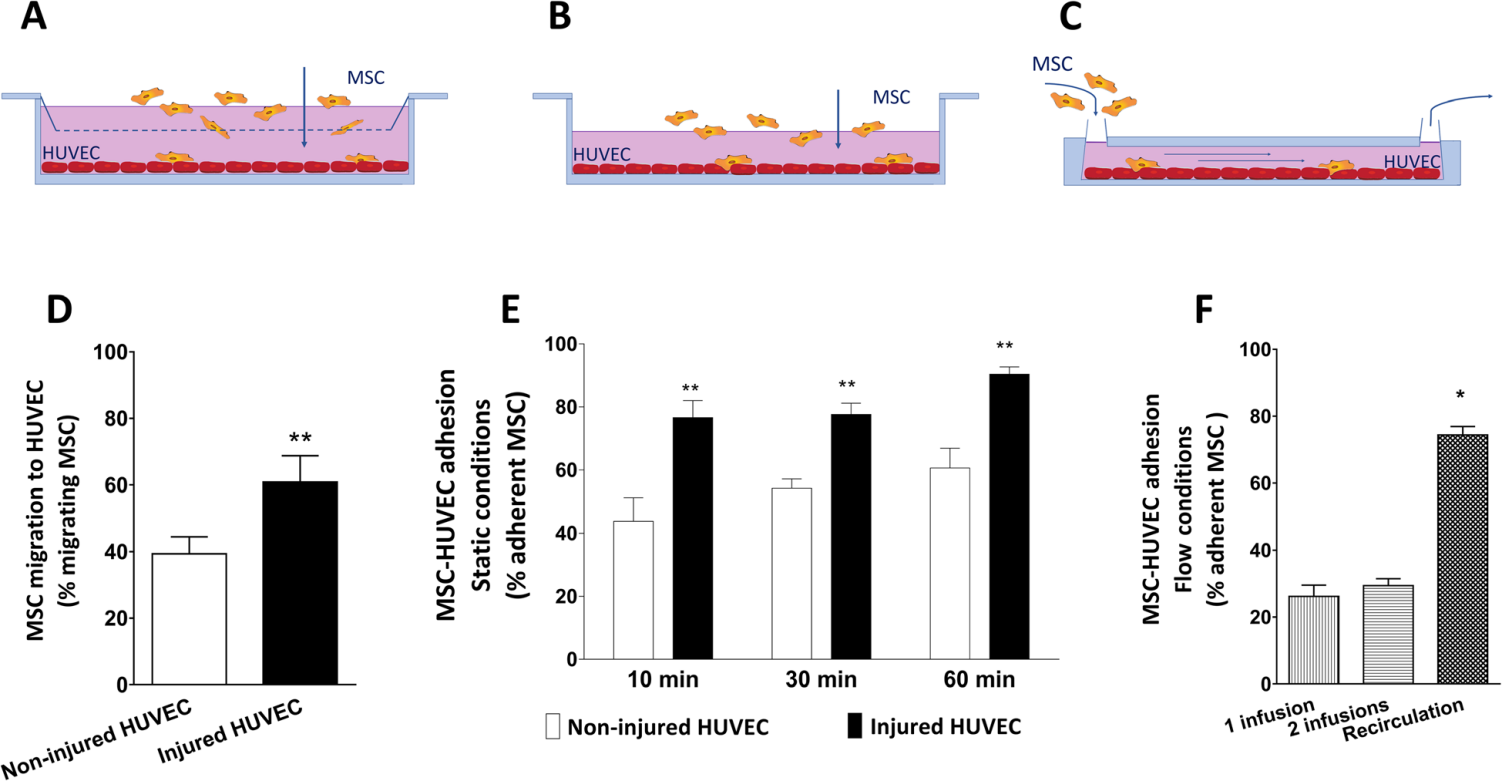
Migration and adhesion of MSC. **a** Migration of MSC is assessed by measuring the percentage of MSC able to migrate through a porous membrane towards (injured) HUVEC. **b** Adhesion of MSC to HUVEC in static conditions. MSC are added on a confluent monolayer of HUVEC, and the percentage of MSC which adhered after 10, 30, and 60 min is assessed by flow cytometry. **c** Adhesion of MSC to HUVEC in flow conditions. HUVEC are grown and injured in a flow chamber. MSC were infused 1 time or 2 times during flow or 1 time and recirculated for 10 min. The percentage of MSC which adhered was assessed by flow cytometry. **d** MSC showed an increased migratory capacity towards injured HUVEC compared to non-injured HUVEC. **e** MSC show increased adhesion to injured HUVEC compared to non-injured HUVEC. **f** MSC showed 28% adhesion capacity to injured HUVEC during flow conditions after one or two times infusion. Recirculation of MSC yielded increased adhesion of MSC to injured HUVEC during flow conditions. Significance of the comparison between 1 time infusion and recirculation is shown (*n* = 5). Results are shown as mean ± SD. **p value < 0.01; *p value < 0.05

MSC migratory capacity towards endothelial cells was tested in a transwell system where MSC were added to the upper well and HUVEC grown to confluence in the lower well (Fig. 1a). After 6 h, 39% of MSC migrated towards non-injured HUVEC. Exposure of HUVEC to hypoxia and reoxygenation led to the migration of 61% of the added MSC towards HUVEC (Fig. 1d).

The capacity of MSC to adhere to HUVEC was tested in static conditions, incubating MSC on a monolayer of HUVEC for 10, 30, and 60 min (Fig. 1b). MSC showed an increased adhesion capacity to injured HUVEC compared to non-injured HUVEC at all time points (Fig. 1e). Importantly, MSC also exhibited adhesion capacity to HUVEC under flow conditions (Fig. 1c). HUVEC were cultured and injured in perfusion slides. Single or double infusions of MSC were administered to the perfusion slides, resulting in the adhesion of less than 30% of the added MSC. The recirculation of MSC in the same system enabled repeated contact of MSC with HUVEC, leading to 74% of the MSC to attach to injured HUVEC (Fig. 1f).

**Fig. 2:**
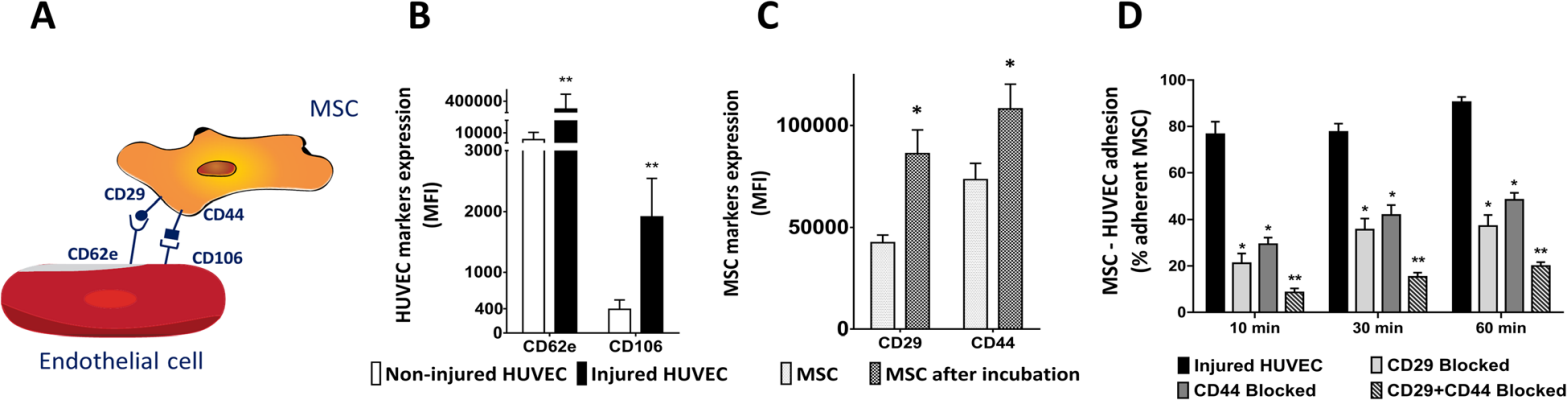
MSC-HUVEC adhesion mechanism. **a** Schematic representation of the molecular mechanism for MSC and HUVEC interaction. **b** The expression of CD62e and CD106 is upregulated on HUVEC membrane after hypoxia and reoxygenation. **c** CD29 and CD44 adhesion molecule expression is increased on the surface of MSC after incubation with injured HUVEC. **d** The blockage of CD29 and CD44 inhibits the adhesion of MSC to injured HUVEC. Significance of the comparison between injured HUVEC and the effect of blocking CD29 and/or CD44 is shown (*n* = 5). Results are shown as mean ± SD. **p value < 0.01; *p value < 0.05

The proposed mechanism for MSC and HUVEC interaction is depicted in Fig. 2a. Upon hypoxia and reoxygenation injury, CD62e and CD106 expression levels on HUVEC membrane were upregulated (Fig. 2b). At the same time, their ligands, CD29 and CD44, were upregulated on the cell membrane of MSC after incubation with injured HUVEC (Fig. 2c). In order to test the importance of these two molecules on MSC-HUVEC interaction, we blocked their binding sites on the surface of MSC. Blockage of CD29and CD44 by blocking antibodies led to the inhibition of MSC adhesion to HUVEC (Fig. 2d) without affecting the survival of MSC assessed by trypan blue (data not shown).

### MSC decrease injury markers on endothelial cells after hypoxia-reoxygenation

**Fig. 3:**
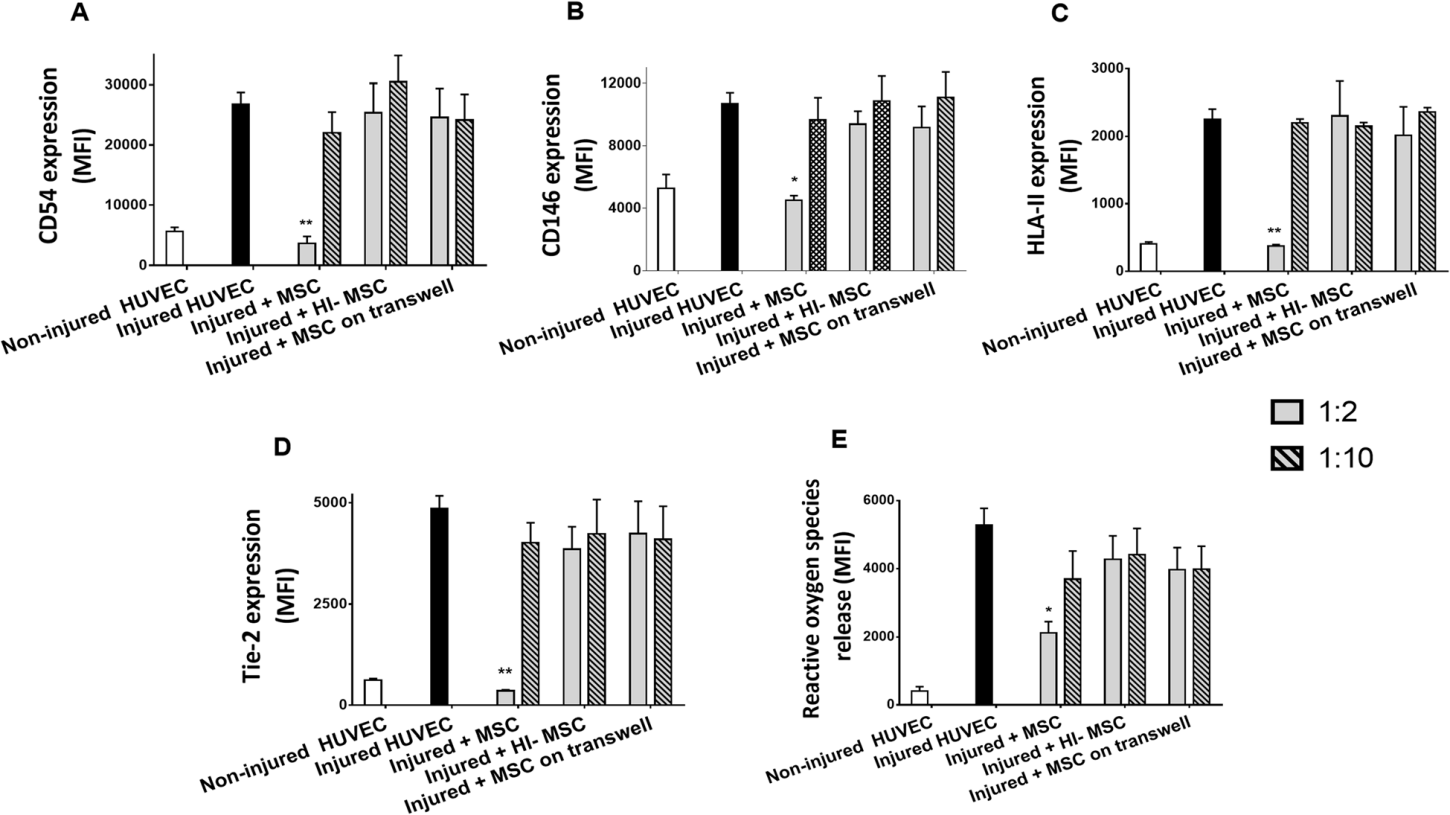
MSC reduce injury markers on injured HUVEC. **a**–**d** The expression of CD54, CD146, HLA-II, and Tie-2 on HUVEC membrane was increased after injury. After 24 h incubation with MSC at a 1:2 ratio, membrane markers were decreased to non-injured levels. No effects of HI-MSC or MSC separated from HUVEC through a transwell were observed. **e** Production of ROS by HUVEC was increased by hypoxia and reoxygenation. After 24 h incubation with MSC at a 1:2 ratio, ROS levels in HUVEC were decreased by 60%. No effects of HI-MSC or MSC incubated through a transwell were observed (*n* = 5). Results are shown as mean ± SD. Significance of the comparison between injured HUVEC and the effect of MSC is shown as **p value < 0.01 and *p value < 0.05

To examine the effect of hypoxia-reoxygenation on endothelial cells, survival and metabolism of HUVEC were studied under hypoxic and inflammatory conditions. Several injury markers were upregulated after this process including adhesion molecules CD54 and CD146, angiogenic receptors such as Tie-2 and VEGFR2, and HLA-II, which is typically upregulated on endothelial cells upon injury (Fig. 3a–d). The release of ROS was increased after injury as well (Fig. 3e).

Injured HUVEC were incubated with either MSC, HI-MSC that lost their ability to secrete factors, or MSC on top of a transwell membrane for 24 h at an MSC-HUVEC 1:2 ratio, or at a 1:10 ratio (Additional file 1D-F). Incubation with MSC at a 1:2 ratio decreased the expression of CD54, CD146, Tie-2, and HLA-II on injured HUVEC to levels close to uninjured HUVEC, while no effect was observed with MSC at a 1:10 ratio (Fig. 3a–d). MSC also decreased ROS levels produced by HUVEC by 60% at a 1:2 MSC-HUVEC ratio (Fig. 3e). When injured HUVEC were incubated with HI-MSC or with MSC separated through a transwell membrane, no effects on HUVEC surface markers or ROS levels were observed.

### MSC improve injured endothelial cell wound healing, barrier, and angiogenic function

**Fig. 4:**
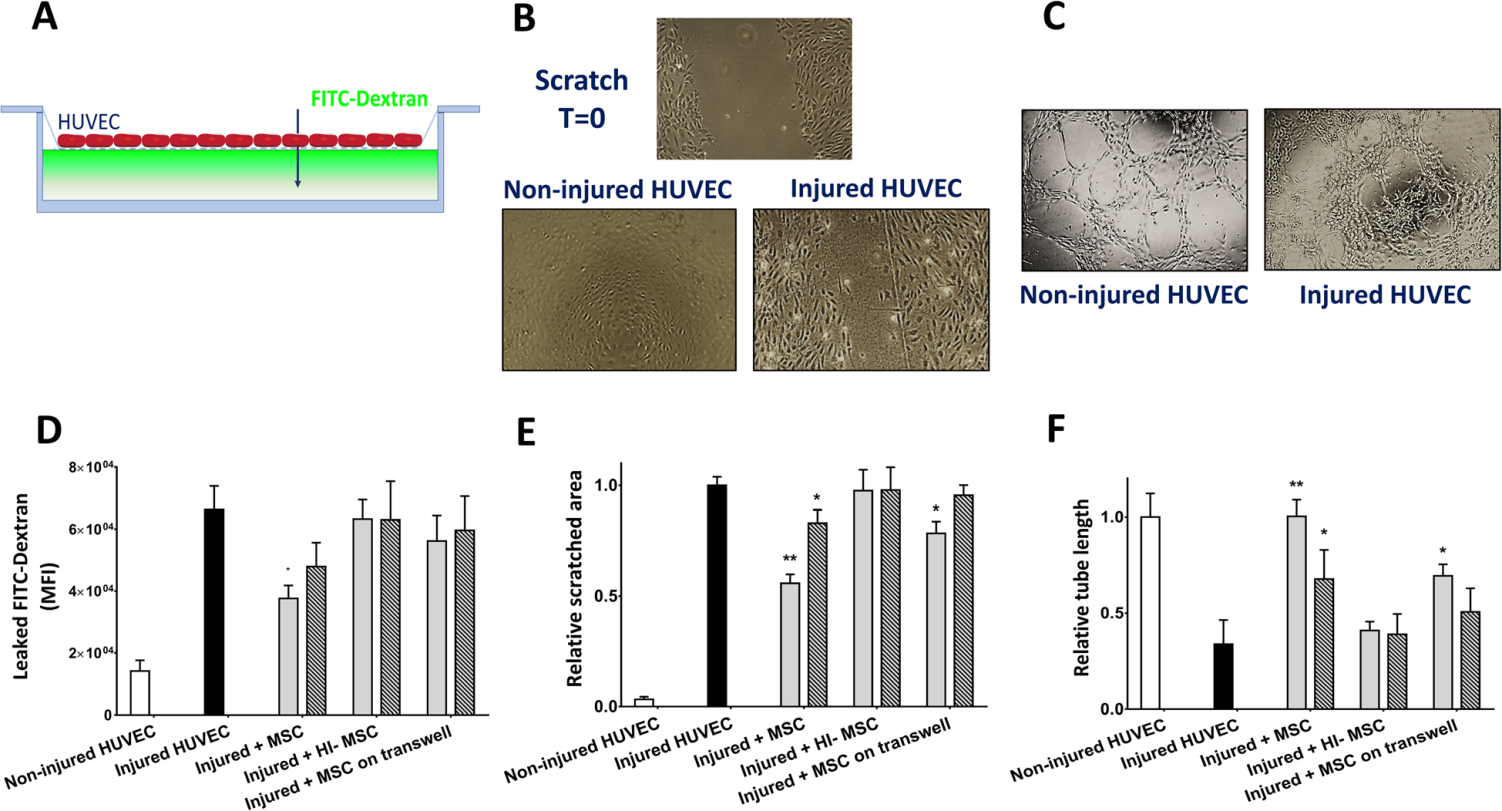
MSC repair wound healing capacity, barrier function, and angiogenic properties of injured HUVEC. **a** HUVEC monolayer permeability was increased after injury. MSC at a 1:2 ratio reduced HUVEC monolayer permeability by 44% after 24 h of incubation. Incubation with HI-MSC or MSC through a transwell had no effect. **b** Hypoxia-reoxygenation injury decreased the wound healing capacity of HUVEC measured by a scratch assay. After 24 h incubation with MSC at a 1:2 ratio, MSC improved HUVEC capacity to close a scratched area by 45%. Secreted molecules by MSC during incubation through a transwell improved injured HUVEC wound healing by 22%. At a ratio of 1:10 MSC-HUVEC, wound healing capacity was improved by 17% by MSC. HI-MSC had no effect at any ratio. **c** The total length of tube-like structures formed by HUVEC was measured to quantify angiogenic capacity. Hypoxia and reoxygenation injury decreased HUVEC angiogenic potential by half. After 24 h of incubation with MSC at a 1:2 ratio, injured HUVEC fully recovered their angiogenic capacity. At a 1:10 ratio, MSC improved angiogenic potential of injured HUVEC by 50%. Secreted molecules by MSC at a 1:2 ratio led to a 48% recovery on injured HUVEC angiogenic potential. HI-MSC had no effect at any ratio. **d** Schematic depiction of endothelial monolayer integrity. Added FITC-conjugated dextran leaks through an injured endothelial monolayer. **e** Visual representation of non-injured (left) and injured (right) HUVEC angiogenic potential. **f** Visual representation of HUVEC wound healing capacity. Top panel: a scratch is made to the endothelial monolayer at time point 0 h. Bottom left panel: non-injured HUVEC completely close the scratch after 6 h. Bottom right panel: injured HUVEC are not able to completely close the scratch after 6 h (*n* = 5). Results are shown as mean ± SD. Significance of the comparison between injured HUVEC and the effect of MSC is shown as **p value < 0.01 and *p value < 0.05

To test the effect of hypoxia-reoxygenation on the permeability of a monolayer of HUVEC, a FITC-labeled dextran leakage assay was used to assess if the dextran leaked through injured HUVEC monolayers (Fig. 4a). Hypoxia-reoxygenation increased endothelial monolayer permeability, as demonstrated by a 3-fold increase in compared to non-injured HUVEC (Fig. 4d). MSC reduced endothelial cell monolayer permeability by 60%, while HI-MSC and MSC separated from HUVEC with a transwell membrane failed to do so. No effects were observed of the lower MSC ratio (Fig. 4d).

The ability of HUVEC to close a scratch in the monolayer, which is a measure of their wound healing capacity, sharply decreased after hypoxia and reoxygenation (Fig. 4b). Incubation with the high MSC ratio was shown to stimulate the capacity of injured HUVEC to close a scratch by 45% whereas the low ratio of MSC improved this capacity by 17%. In addition, MSC incubated through a transwell had a small and dose-dependent effect on the scratch closing ability of HUVEC (Fig. 4e).

Moreover, the angiogenic potential of HUVEC was measured by their capacity to form tubes in vitro, which was decreased by 50% compared to non-injured endothelial cells (Fig. 4c). The addition of MSC at a 1:2 ratio fully restored HUVEC angiogenic potential while MSC at a 1:10 ratio improved this capacity to 50% of the potential of non-injured HUVEC. HI-MSC did not elicit a reparative effect. In addition, the high dose of MSC separated from injured HUVEC via a transwell membrane improved their angiogenic capacity by 48% (Fig. 4f).

### MSC transmigrate through an endothelial monolayer in vitro

**Fig. 5:**
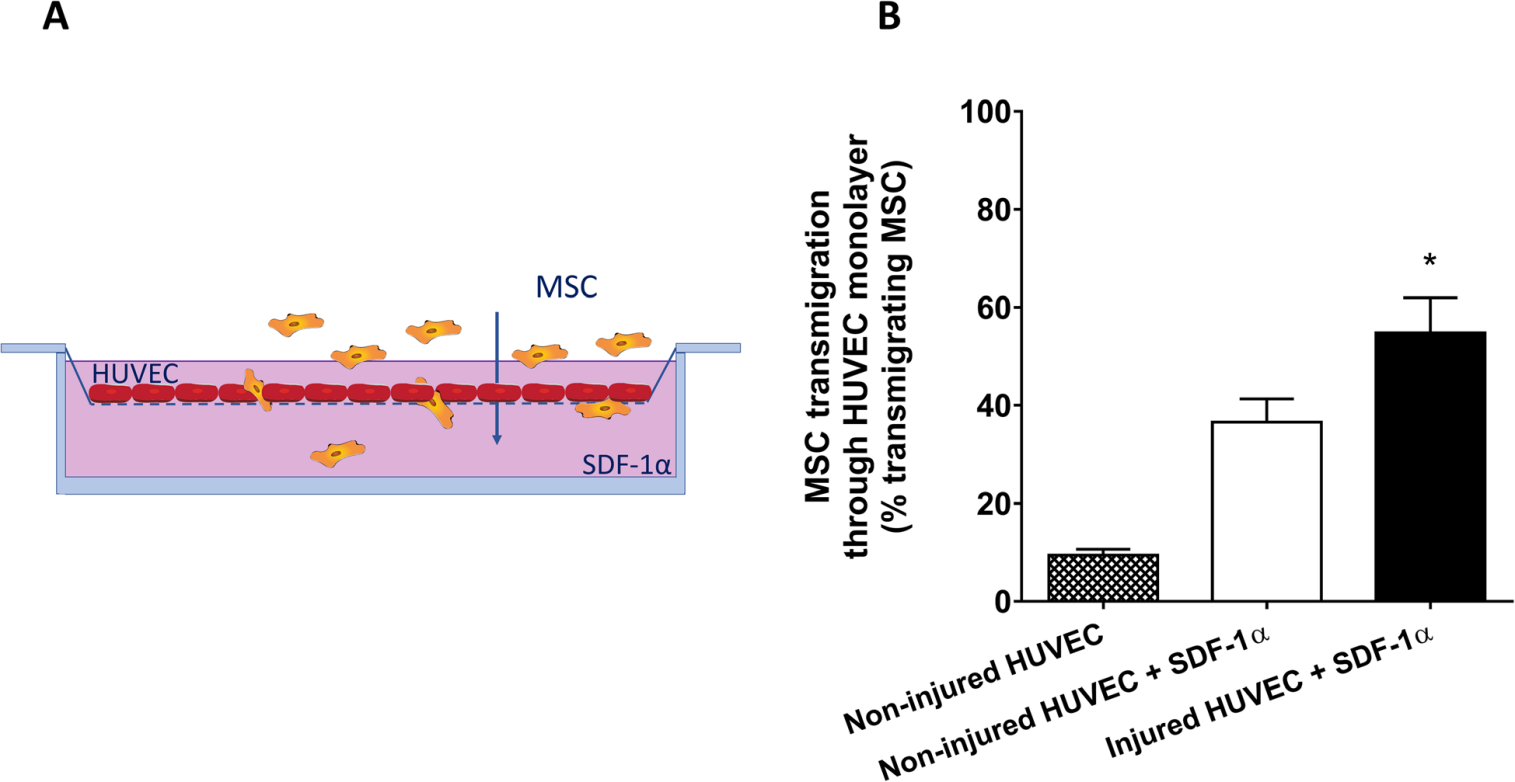
MSC transmigrate through an endothelial monolayer towards kidney injury chemokine SDF-1α. **a** Schematic representation of the transwell assay to assess the capacity of MSC to transmigrate through a monolayer of endothelial cells. **b** MSC showed the capacity to transmigrate through a confluent endothelial monolayer towards SDF-1α. Injury of HUVEC enhanced the transmigration capacity of MSC, resulting in 54% of the added MSC transmigrating through the injured HUVEC monolayer after 6 h (*n* = 5). Results are shown as mean ± SD. Significance of the comparison between injured HUVEC + SDF-1α and non-injured HUVEC + SDF-1α is shown as *p value < 0.05

We went on examining the transmigration of MSC through a HUVEC monolayer as depicted in Fig. 5a, which would potentially allow MSC to provide regenerative signals to tissues underlying the endothelium. MSC did not show migratory capacity through an endothelial cell monolayer after 6 h without a chemotactic signal underneath the endothelial monolayer (Fig. 5b). Nonetheless, MSC were able to migrate through a HUVEC monolayer after adding SDF-1α to the lower well of the transwell system. After 6 h, 38% of the added MSC migrated through a monolayer of HUVEC (Fig. 5b). The migratory capacity of MSC was enhanced when HUVEC were injured. These in vitro experiments indicate that MSC have the potential to pass the endothelium and migrate into tissues towards gradients of chemotactic signals.

## Discussion

In this manuscript, we described the reparative effect of MSC on HUVEC damaged by hypoxic and inflammatory conditions. The combination of physical and paracrine interaction of MSC with injured HUVEC proved to be the key in restoring the wound healing and angiogenic potential of HUVEC as well as for the decrease in HUVEC oxidative stress and other injury markers. The migration of MSC towards injured HUVEC, their adhesion, and subsequent transmigration through a HUVEC monolayer, in which CD29 and CD44 are key mediators, suggest that MSC are able to interact with endothelium after ischemia and reperfusion injury during the donation and transplantation procedures. The suppression of hypoxia-reoxygenation effects on HUVEC adhesion markers, the decrease in oxidative stress levels, and the complete recovery of injured HUVEC angiogenic potential suggest that MSC have the capacity to restore endothelial function.

We established a hypoxia and reoxygenation model in vitro to mimic endothelial inflammatory injury [1, 27, 28]. The use of HUVEC was chosen based on their proven value for in vitro endothelial hypoxia-reoxygenation injury studies [29, 30]. However, we realize that complementary studies on the regenerative effect of MSC on tissue-specific endothelial cells, such as microvascular endothelial cells in kidneys, will be necessary to confirm our results in organ-specific in vitro models.

Renal endothelial injury is correlated with kidney function loss [31, 32]. In addition, in human renal IRI after donation and transplantation, endothelial permeability is increased and results in delayed graft function [33]. Endothelial injury is associated with the release of pro-inflammatory factors and increased expression of adhesion molecules on endothelial cells after IRI, promoting immune cell recruitment and infiltration [34–37]. Our data show that MSC decrease the expression of injury markers on injured HUVEC and reduce oxidative stress levels and endothelial permeability, suggesting that MSC could repair IRI effects on endothelium in a kidney transplantation setting.

The cytokines and adhesion molecules that are upregulated upon endothelial injury can also promote MSC migration and adherence to endothelial cells [38, 39]. In our model, we used SDF-1α as a chemoattractant as it is secreted under pro-inflammatory conditions. Moreover, MSC express the receptor for SDF-1α, CXCR4 [38]. We showed that MSC are able to migrate towards injured HUVEC and adhere in an inflammatory microenvironment under static and flow conditions. The latter is of relevance because MSC infused in the bloodstream have to interact with endothelial cells as they are transported by peripheral blood flow. We show that CD62e and CD106 expression levels, among others, are increased on HUVEC after hypoxic injury, and their ligands CD29 and CD44 [40, 41] are increased on MSC membrane after incubation with injured HUVEC. Blocking these two markers on the MSC surface resulted in a great inhibition of MSC adherence, suggesting that these molecules play a key role on MSC-HUVEC interaction [42, 43]. Enhancing the expression of these two proteins during MSC in vitro culture could improve MSC delivery to endothelial cells [44]. In addition, targeted delivery of MSC to the injured organ would be useful to improve their interplay with the injured tissue. Direct infusion through the renal artery and delivery during normothermic machine perfusion are plausible options to deliver cells specifically to the kidney and ensure MSC interaction with damaged renal endothelium during organ preservation [24].

The fate of MSC after delivery to the kidney is currently unclear, and there is little evidence about the invasion of MSC in tissues underlying the endothelium. Several publications show that after IV infusion, MSC die and are not detectable in the target organ within 24 h [13, 45, 46]. Targeted infusion of MSC through the renal artery ensures high delivery efficiency of MSC to the kidney. However, the fate of MSC administered in this manner to the kidney is unclear. We previously reported a sharp decline in the number of MSC present in kidney biopsies at 2 weeks after administration of MSC [23]. It is expected that MSC would not stay at the luminal side of microcapillaries, but that these cells are either removed by innate immune cells or migrate to other sites [13, 47]. Previous reports suggested that MSC engraft in tubular and glomerular structures after infusion via the renal artery [15, 46], but these results remain controversial and have not been confirmed in recent studies. We observed in our in vitro experiments that MSC possess the capacity to migrate through endothelial cell layers which would suggest they are able to invade tissues. The enhanced migratory capacity of MSC after exposure of HUVEC to hypoxia and TNF-α described in our study might be due to both the upregulation of the molecules involved in MSC-HUVEC physical interaction, in particular CD29-CD62e and CD44-CD106, and to the increased permeability of the endothelial monolayer after hypoxic and inflammatory damage. However, it has to be noted that in vitro experiments are performed at the most optimal conditions for cell activity and these are usually far from physiological conditions (concentration of triggering molecules, composition of the medium, etc.). Therefore, more studies are necessary to examine whether MSC harbor this property in vivo, which could be achieved for instance by real-time intravital tracking of MSC delivered to an animal model, visually unraveling the fate of MSC after infusion [48].

Likewise, the main mechanism behind the regenerative effects of MSC has been widely debated. In this study, we show that the soluble factors secreted by MSC can promote the regeneration of injured HUVEC. However, the full regenerative potential of MSC required both physical and paracrine interaction between MSC and HUVEC. In this case, the in vitro setting is restricting many of the interactions occurring in vivo, which may be responsible for the actual observed MSC effect, as previously described [13].

## Conclusion

We conclude that MSC harbor the capacity to control the damage associated with hypoxia-reoxygenation injury on endothelial cells provided that they may interact physically and through secreted molecules. Our results suggest that MSC are a valuable therapeutic option to repair IRI, and future studies will reveal whether and how MSC can be applied to repair organs prior to and after transplantation.

## Supplementary information

**Additional file 1.** Hypoxia reoxygenation model and MSC-HUVEC interaction models. A, oxygen levels drop immediately to 0% after adding catalase and glucose oxidase to the culture medium. After 24 h in a hypoxia incubator, the oxygen percentage in the medium remained 0% until contact with air was re-established. B and C, HUVEC did not die nor become apoptotic after culture in hypoxia and upon reoxygenation in the presence of TNF-α. D, MSC co-cultured with injured HUVEC allowing physical interaction and soluble factors secretion. E, Heat-inactivated (HI)-MSC co-cultured with injured HUVEC enabling only physical interaction, as HI-MSC lost their ability to secrete growth factors and cytokines. F, MSC co-cultured with injured HUVEC in a transwell system to avoid cell-to-cell contact but allow interaction through secreted molecules.

## Data Availability

The data that support the findings of this study are available from the corresponding author upon reasonable request.

## Abbreviations

EGM-2: Endothelial growth medium 2
HUVEC: Human umbilical vein endothelial cells
IRI: Ischemia-reperfusion injury
IV: Intravenous
MSC: Mesenchymal stromal cells
SDF-1α: Stromal cell-derived factor 1α
TNF-α: Tumor necrosis factor-α
